# Predicting Driver Behavior during the Yellow Interval Using Video Surveillance

**DOI:** 10.3390/ijerph13121213

**Published:** 2016-12-06

**Authors:** Juan Li, Xudong Jia, Chunfu Shao

**Affiliations:** 1 MOE Key Laboratory for Urban Transportation Complex Systems Theory and Technology, Beijing Jiaotong University, Beijing 100044, China; cfshao@bjtu.edu.cn; 2 Civil Engineering Department, California State Polytechnic University, Pomona, CA 91768, USA; xjia@cpp.edu

**Keywords:** video surveillance, driver behavior, signalized intersection, sequential logit model

## Abstract

At a signalized intersection, drivers must make a stop/go decision at the onset of the yellow signal. Incorrect decisions would lead to red light running (RLR) violations or crashes. This study aims to predict drivers’ stop/go decisions and RLR violations during yellow intervals. Traffic data such as vehicle approaching speed, acceleration, distance to the intersection, and occurrence of RLR violations are gathered by a Vehicle Data Collection System (VDCS). An enhanced Gaussian Mixture Model (GMM) is used to extract moving vehicles from target lanes, and the Kalman Filter (KF) algorithm is utilized to acquire vehicle trajectories. The data collected from the VDCS are further analyzed by a sequential logit model, and the relationship between drivers’ stop/go decisions and RLR violations is identified. The results indicate that the distance of vehicles to the stop line at the onset of the yellow signal is an important predictor for both drivers’ stop/go decisions and RLR violations. In addition, vehicle approaching speed is a contributing factor for stop/go decisions. Furthermore, the accelerations of vehicles after the onset of the yellow signal are positively related to RLR violations. The findings of this study can be used to predict the probability of drivers’ RLR violations and improve traffic safety at signalized intersections.

## 1. Introduction

Intersections are one of the major traffic safety challenges to drivers. In 2012, 2850 roadway fatalities and 680,000 injury crashes were intersection-related in the United States [1]. A total of 22% of fatal crashes and 45% of serious crashes occurs at intersections. One of the major causes of intersection-related crashes is drivers’ inability to make correct decisions at intersections. Therefore, it is necessary to predict drivers’ behaviors, assist drivers in making correct decisions when they approach intersections, and reduce crashes at signalized intersections [2].

### 1.1. Stop/Go Decisions

When a vehicle approaches a signalized intersection, its driver needs to make a stop/go decision at the onset of the yellow signal. In this case, an incorrect decision may result in crashes or red light running (RLR) violations [3,4,5,6,7,8,9,10,11]. When a driver makes a conservative “stop” decision, the vehicle may stop abruptly with a relative short distance to the stop line at the onset of the yellow signal, which may cause a rear-end crash. Alternatively, when a driver has an aggressive ”go” decision, the vehicle may pass through the intersection directly with a relative long yellow-onset distance, which may lead to an RLR violation and a right-angle collision. Drivers’ uncertain actions increase safety concerns at signal-controlled intersections. Therefore, it is essential to study drivers’ behaviors during yellow intervals at signalized intersections. According to previous studies [12,13,14,15], a road segment where drivers make decisions at the onset of the yellow signal can be specified as a “cannot go” region, a “cannot stop” region, a “dilemma zone” or an “option zone”.

**Figure 1:**
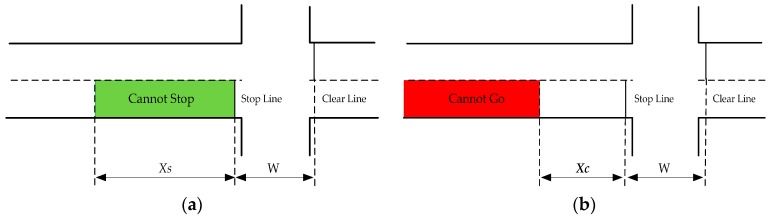
“Cannot Stop” and “Cannot Go” regions. (**a**) “Cannot Stop” region; (**b**) “Cannot Go” region. Note: $X_{s}$ is the minimum distance for the vehicle to come to a complete stop; $X_{c}$ is the maximum distance to the stop line in which the vehicle can pass through the intersection safely and completely; *W* is the width of the intersection.

If a driver decides to stop at the onset of a yellow signal, he/she would decelerate. The stopping distance of the vehicle is the distance traveled during the perception-reaction time and the braking distance, which can be described as follows in Equation (1) (assuming the effect of grade is ignored):

(1) $$X_{s}=v\times prt+\frac{v^{2}}{2d}$$

where $X_{s}$ is the minimum distance for the vehicle to come to a complete stop; v is the vehicle’s approaching speed in m/s; *prt* is the perception-reaction time in seconds; and d is the constant deceleration rate in m/s^2^. As depicted in Figure 1a, if a vehicle is positioned within the region between the stop line and distance $X_{s}$ at the onset of the yellow signal, it would not be expected that the driver can stop completely and comfortably. This region is termed as a “cannot stop” region.

If a driver decides to drive into an intersection at the onset of the yellow signal, he/she will accelerate or maintain the current speed to cross the intersection. The distance for the vehicle to safely pass the intersection can be represented as follows in Equation (2):

(2) $$X_{c}=v\times (Y+R)+\frac{a\times {\left(Y+R-prt\right)}^{2}}{2}-(W+L)$$

where $X_{c}$ is the maximum distance to the stop line in which the vehicle can pass through the intersection safely and completely; v is the vehicle’s approaching speed in m/s; *prt* is the perception-reaction time in seconds; a is the vehicle’s acceleration rate in m/s^2^; *Y* is the yellow interval; *R* is the red clearance interval in seconds; *W* is the width of the intersection; and *L* is the length of the vehicle in m. If the vehicle is at a position beyond $X_{c}$ at the onset of the yellow signal, the intersection is not to be cleared when the signal turns to red. The region upstream of the distance of $X_{c}$ is termed as a “cannot go” zone, as indicated in Figure 1b.

**Figure 2:**
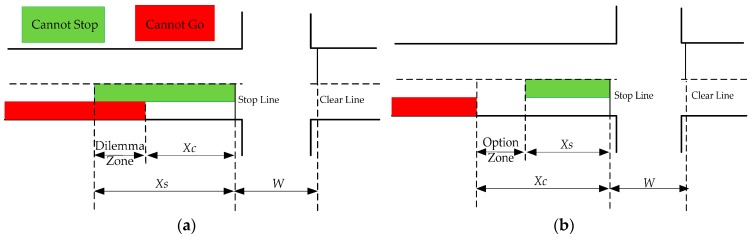
Dilemma zone and option zone. (**a**) Dilemma zone; (**b**) Option zone.

The dilemma zone and option zone are the areas determined by the relationship between the distance $X_{s}$ and $X_{c}$. These areas are hazardous portions of an intersection, and decisions made in these areas could result in traffic conflicts or RLR violations. The dilemma zone shown in Figure 2a is the overlapping area of the “cannot stop” region and the “cannot go” region. In this overlapping case, $X_{s}>X_{c}$, the driver neither stops comfortably before the stop line nor crosses the intersection safely. These drivers tend to decelerate or accelerate unsafely, which could result in rear-end collisions or RLR violations. On the other hand, the option zone shown in Figure 2b is a gap between the “cannot stop” region and the “cannot go” region. In this gap, $X_{s}<X_{c}$, drivers either stop before the stop line comfortably or clear the intersection safely during their yellow interval. If two successive drivers make opposite stop/go decisions, a rear-end conflict may take place.

Due to uncertainty of stop/go decisions and potential conflicts at intersections, many researchers have focused on the study of drivers’ behaviors during yellow intervals at signalized intersections. Gazis [16] pointed out the problem of yellow intervals in 1960. Herman et al. [17] and Crawford et al. [18] did the pioneering work of defining a dilemma zone based on the distance of vehicles to the stop line. Zegeer [19] specified a dilemma zone by the probability of drivers’ “stop” decisions from practice. He defined the zone as an area where 10% and 90% of drivers would choose to stop at the onset of the yellow signal and consider this area as a Type II dilemma zone. Sheffi and Mahmassani [20] modeled driver behavior at signalized intersection as a binary decision (stop or go) and used a probit calibration routine to estimate model parameters. Liu et al. [21] utilized an ordered-probit model to describe the impacts of contributory factors in drivers’ stop/go decisions. Lu et al. [22] used a regression model to depict the correlation between traffic parameters and drivers’ behavior during yellow intervals, and found the number of drivers with “go” decisions are associated with traffic volume. Ohlhauser et al. [23] applied a logistic model to analyze the effect of drivers’ age, driving distraction, and time to the stop line on the likelihood of stopping during yellow intervals. Savolainen et al. [24] used a panel data random probability method to estimate the impacts of factors on drivers’ behaviors during the yellow interval, such as yellow interval duration, warning flasher, and camera enforcement.

### 1.2. RLR Violation

Incorrect “go” decisions may result in RLR violations and crashes at signalized intersections [3,4,11,25]. According to a report of the Ministry of Public Security, China, during 10 months of 2010, 789 fatal crashes and 4227 severe injury crashes were caused by RLR violations [26]. Previous studies demonstrate that RLR violations have statistical correlations with the following factors:

(1) Driver and vehicle characteristics: Male, young, and unbelted drivers without passengers are prone to RLR violations [27]. These drivers have worse driving records than non-RLR drivers [28]. Furthermore, drivers in small and old vehicles are more likely to run red lights. In addition, vehicles with high approaching speed have a positive relationship with RLR violations [22].
(2) Intersection geometric design: Larger intersections and wider approaches are highly associated with increased RLR violations rates [29].
(3) Signal timing: Drivers are more likely to run red lights with a shorter cycle length or yellow interval less than 3.5 s [30].
(4) Traffic conditions: Traffic volumes and the presence of pedestrians and bicycles are two important influencing factors of RLR violations [31].
(5) Strategies of enforcement: Red light cameras have positive effects on reducing the possibility of RLR violations, while the flashing signal increases the frequency of RLR violations [29].

### 1.3. Objectives and Advantages

There are many previous studies with a focus on identifying the factors that influence drivers’ stop/go decisions and RLR violations. Some of these studies adopted machine learning techniques to predict drivers’ behaviors. Elmitiny [32] used a classification tree model to predict RLR violations and identified the major factors resulting in RLR violations. Jahangiri et al. [31,33] employed the Support Vector Machine (SVM) and Random Forest (RF) technique to develop driver behavior models and demonstrated that the approaching speed of vehicles is a valuable predictor of driver behavior.

Previous studies also used regression models to estimate driver behavior [34,35,36]. These models obtained the explanatory factors contributing to driver behavior and interpreted the marginal effects of these factors. For example, Papaioannou et al. [37] classified drivers into three categories according to their driving aggressiveness. A logistic regression model was utilized to explain drivers’ behaviors. It was found that the contributing factors were different for different categories of drivers. Yan et al. [38] used binary logit models to evaluate the impacts of pavement marking on drivers’ stop/go decisions and found that this countermeasure could help drivers make correct stop/go decisions and reduce RLR violations.

The previous methods worked well for stop/go decisions and RLR violations separately, but they did not consider the correlation between them. The objective of this study is to develop a sequential logit model to analyze the relationship between drivers’ stop/go decisions and RLR violations. There are two advantages of this study. First, a video detection algorithm is used to accurately extract drivers’ behaviors in the case of yellow intervals. Secondly, a sequential logit model is applied to depict the relationship of drivers’ stop/go decisions and RLR violations. This study provides a useful method for predicting drivers’ behaviors during yellow intervals and helps drivers avoid RLR violations.

The rest of this paper is organized as follows. Section 2 introduces a conceptual framework and a method for modeling drivers’ behaviors. Section 3 presents a statistical analysis based on real-time traffic data. Section 4 presents the results and a detailed discussion. Finally, Section 5 concludes the paper by summarizing the major findings of the study.

## 2. Methodology

### 2.1. Conceptual Framework

The study analyzes driver behavior during the yellow interval through a Vehicle Data Collection System (VDCS) and has the following tasks:

(1) Detecting vehicles that approach intersections and recording the trajectories of the vehicles in video streams.
(2) Recording signal operations and traffic parameters while vehicles are approaching intersections. These parameters include the vehicle distance to the stop line, approaching speed, and acceleration.
(3) Analyzing the relationship between drivers’ stop/go decisions and RLR violations.

**Figure 3:**
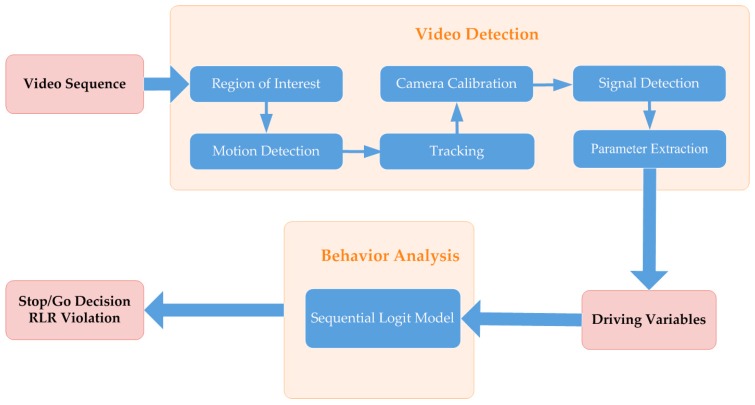
Conceptual framework for RLR violation analysis.

Figure 3 presents a conceptual framework for analyzing RLR violations. Within this framework, vehicle position, type, distance to the stop line, approaching speed at the onset of the yellow signal, and acceleration during the yellow interval, as well as drivers’ stop/go decisions and RLR violations are autonomously extracted from recorded video streams. A sequential logit model is then utilized to analyze the reasons for RLR violations.

### 2.2. Vehicle Data Collection System (VDCS)

The Vehicle Data Collection System (VDCS) is used to capture drivers’ behavior data such as drivers’ stop/go decisions, RLR violations, vehicle distances to the stop line, approaching speed and acceleration during the yellow interval. This VDCS, developed in C++ with the OpenCV package, includes six modules: the region of interest (ROI) module, the motion detection module, the tracking module, the camera calibration module, the signal detection module, and the parameter extraction module. With the help of the VDCS, lanes are first selected by the ROI module. Moving vehicles in the selected or target lanes are then detected by the motion extraction module. Vehicle trajectories are further extracted by the Kalman Filter (KF) algorithm in the tracking module. Vehicle displacements are further tracked by the camera calibration module through the transformation of the camera’s image units to physical coordinates for vehicles. The changes in signal indications are identified and recorded in the signal detection module based on the colors of the signal lamps. Driver behavior data are extracted and synchronized with the signal indications in the parameter extraction module. Key algorithms used in the VDCS are described as follows.

#### 2.2.1. Motion Detection

Moving vehicles are extracted from image sequences by using the background subtraction technology [39] in the motion detection module. A Gaussian Mixture Model (GMM) is widely used for the detection due to its adaptability to detect multiple backgrounds. Every pixel value is modeled as a mixture of Gaussian distributions, and the distribution with sufficient and consistent evidence would become the background colors [40]. The GMM uses the probability of each pixel to determine whether the pixel belongs to the background or foreground. The GMM is denoted as follows in Equation (3):

(3) $$P(X_{t})=\sum_{k=1}^{K}\omega_{k,t}\times \eta (X_{t},\mu_{k,t},\sum_{k,t})$$

where $\omega_{k,t}$ is the weight parameter of the *k*th Gaussian distribution and $\eta (X_{t},\mu_{k,t},\sum_{k,t})$ is the probability density function of *k*th Gaussian distribution. Background colors are determined by the *B* highest probable colors, which stay long and static in the image sequences. A pixel is checked against the background distributions. If the pixel does not match any background distribution, the pixel is identified as foreground, otherwise the pixel is considered as background. The first matched distribution can be updated as follows in Equation (4):

(4) $$\left\{ \begin{array}{l} \omega_{k,t}=(1-\alpha )\omega_{k,t-1}+\alpha (M_{k,t})\\ \mu_{k,t}=(1-\rho )\mu_{k,t-1}+\rho X_{t}\\ \sum_{k,t}=(1-\rho )\sum_{k,t-1}+\rho {\left(X_{t}-\mu_{k,t}\right)}^{T}(X_{t}-\mu_{k,t})\\ \rho =\alpha \eta (X_{t},\mu_{k,t},\sum_{k,t}) \end{array}\right.$$

where α is the learning rate; *M_k,t_* is 1 for the matching distribution and 0 for the remaining distributions; ρ is the learning rate for parameters.

When vehicles approach signalized intersections, they may opt to stop during yellow intervals. If the stopped vehicles stay stationary for a long period, these stopped vehicles would be treated as a part of background. As such, they cannot be detected as moving objects. To correctly recognize these temporarily stopped vehicles, the GMM is enhanced in this study to extend the time before the stopped vehicles are merged into background. At the stage of parameter updating, only the pixels belonging to their previous static region are updated. In doing so, the computing speed is increased and the chance of pixels being treated as background is reduced. At the stage of background estimation, an attenuation coefficient is added to reduce the possibility that the pixels of the vehicles are treated as background. At the stage of foreground segmentation, the average weight, which delays the time of the pixels of the vehicles being merged into the background distributions, is adopted. These enhancements not only reduce computation redundancy but also effectively solve the problem of treating temporarily stopped vehicles as background.

#### 2.2.2. Vehicle Tracking

The vehicle tracking module identifies moving objects in successive frames [41]. The module uses a box to track a vehicle in video streams. This box represents the contour of a vehicle with minimum area [42]. The position of the vehicle is recorded as the centroid point of the box. The Kalman Filter (KF) is used to obtain a vehicle’s trajectory as follows in Equations (5) and (6) [43]:

(5) $$x_{t}=A\times x_{t-1}+B\times u_{t}+w_{t}$$

(6) $$z_{t}=H\times x_{t}+v_{t}$$

where $x_{t}$ is the state vector; $z_{t}$ is the measurement vector; $u_{t}$ is the control vector; A and H are the state transition matrix and the measurement matrix, respectively; and $w_{t}$ and $v_{t}$ are the random variables representing the process and the measurement noise, respectively. In the VDCS, Equation (7) represents the vehicle’s position at time *t*:

(7) $$x_{t}=(p_{x,t},p_{y,t})$$

The current position of a vehicle is predicted based on the coordinates of the vehicle in previous frames. With the predicted vehicle position, the VDCS searches for the target vehicle. If the target vehicle is found, the tracking template $T_{t}$ will be updated by the target vehicle as follows in Equation (8):

(8) $$T_{t}=(1-\beta )T_{t-1}+\beta x_{t}$$

where β is a learning parameter. This improved approach reduces the searching time and makes the tracking algorithm more robust.

#### 2.2.3. Transformation of the Coordinate System

The camera calibration module transforms 2-D image coordinates (pixels) in the UV system to 3-D coordinates in the XYZ system [44]. The core of this transformation is to calibrate the camera’s intrinsic and extrinsic parameters. Four physical reference points within an intersection are selected to set the eight parameters for the transformation matrix. After the camera calibration, the physical coordinates of vehicles can be calculated from their image coordinates.

#### 2.2.4. Signal Detection

The signal detection module captures the changes in signal indications by setting a rectangle around the signal indications on recorded video frames. The Hough transformation method is employed to detect the signal indications as circles in the rectangle. When the signal indications change their colors, the VDCS can detect the changes. At the onset of the yellow signal, the VDCS triggers the parameter extraction module to obtain the traffic parameters and identify stop/go decisions and RLR violations.

#### 2.2.5. Traffic Parameter Extraction

The parameter extraction module extracts traffic parameters and driver behavior data from the trajectories of vehicles. With a video stream feeding the VDCS at the rate of 30 frames per second, vehicle speed can be calculated by checking the displacement of the vehicle in two successive frames. Similarly, acceleration is obtained by the speed change of two successive frames. The traffic parameters (such as vehicle position, speed, and acceleration) and the driver behavior data (such as drivers’ stop/go decisions, RLR violations, vehicle distances to the stop line) are further synchronized with signal indications. In doing so, a driver’s stop/go decision at the onset of the yellow signal can be detected based on the speed changes of the vehicle during the yellow interval. RLR violations can also be identified by checking the time duration of a vehicle’s crossing stop line and the onset of the red signal indication.

### 2.3. Behavior Analysis

#### 2.3.1. Logit Model

Logit models are widely used in transportation research because of their closed-form formula and explicit interpretation. Assuming *Y* is a binary response variable in a binary logit model, *Y* = 1 if the response is yes, otherwise *Y* = 0. The probability of the response (*Y* = 1) can be estimated by the following Equation (9):

(9) $$\pi_{i}=P(Y_{i}=1|X_{i}=x_{i})=\frac{e^{g(x)}}{1+e^{g(x)}}$$

where g(x) is the utility function of the explanatory factors, which can be expanded as follows in Equation (10):

(10) $$g(x)=\log(\frac{\pi_{i}}{1-\pi_{i}})=\beta_{0}+\beta_{1}x_{i1}+...+\beta_{k}x_{ik}$$

where $x_{ik}$ is the value of variable k for stage i and $\beta_{k}$ is the coefficient of variable k.

#### 2.3.2. Model Structure

**Figure 4:**
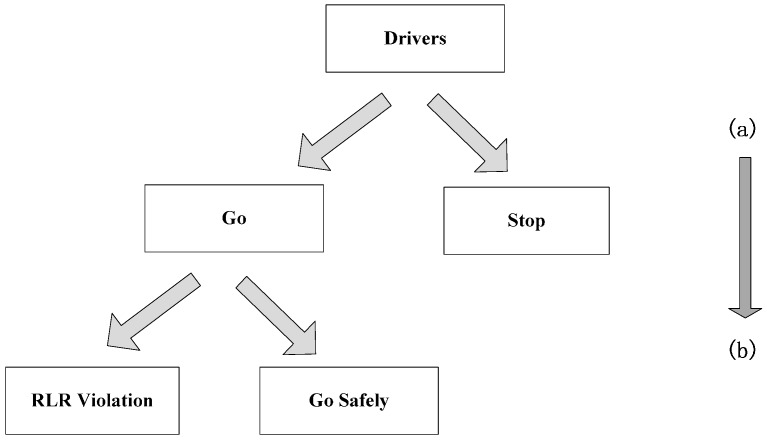
Conceptual Representation of the Sequential Logit Model: (**a**) First Model—the Stop/go Decision Model; and (**b**) Second Model—the RLR Model.

In this study, a sequential binary logit model is used to describe the sequence of drivers’ behaviors [45]. At the onset of the yellow signal, a driver first makes a stop/go decision and then crosses the intersection. The sequential logit model simulates the two-step decision process. As shown in Figure 4, the top-level logit model (Model 1) considers drivers’ stop/go decisions, while the bottom-level logit model (Model 2) traces vehicles to see whether they run red lights. In mathematical terms, Model 1 describes drivers’ “go” (*Y* = 1) and “stop” (*Y* = 0) decisions at the onset of the yellow signal, while Model 2 checks the results of “go” decisions, that is, RLR violations (*Y* = 1) or passing through intersections safely (*Y* = 0).

In Model 1, the likelihood of a stop decision is defined as follows in Equation (11):

(11) $$P(\mathrm{Stop})=P_{1}$$

In Model 2, the probability of an RLR violation can be calculated as follows in Equation (12):

(12) $$P(\mathrm{RLR})=P(\mathrm{RLR}|\mathrm{Go})P(\mathrm{Go})=P_{2}(1-P_{1})$$

where $P_{i}$ denotes the probability of *Y* = 1 of Model i.

## 3. Experimental Evaluation

**Figure 5:**
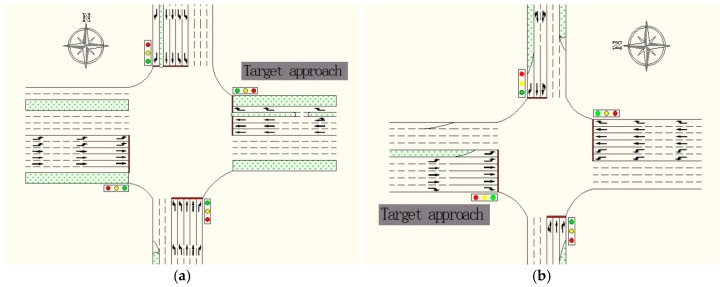
Graphical illustration of the intersections. (**a**) Naoshikou Street @ Xuanwumen West Street; (**b**) Zaojunmiao Road @ Xueyuan South Road.

Field surveys were conducted in Beijing, China, in January 2014. The traffic data were collected at two intersections (the intersection of Naoshikou Street @ Xuanwumen West Street, and the intersection of Zaojunmiao Road @ Xueyuan South Road). The summary of the lane configurations and traffic data at these two intersections is shown in Table 1, along with the graphical illustrations in Figure 5. The yellow interval is four seconds, and the posted speed limits of all streets are 60 km/h.

Two cameras were installed at each intersection to record video streams simultaneously during the survey time. One camera was used to record vehicles approaching the intersection. The other camera was used to record signal indication. The video streams were set at 30 frames per second.

**Figure 6:**
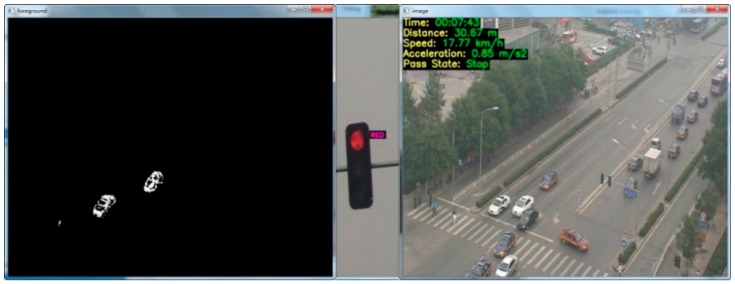
Snapshot of the VDCS.

The VDCS was used to process the recorded videos streams and extract traffic parameters associated with yellow intervals. Figure 6 shows a snapshot of the VDCS. When the system detects a signal turning to yellow, the traffic parameter extraction module is triggered to record a vehicle’s position and time and to extract driver behavior such as a vehicle’s distance to the stop line, approaching speed, acceleration/deceleration, and stop/go decision.

A total of 1086 vehicles were extracted by the VDCS. Among them, 599 vehicles were captured at the intersection of Naoshikou Street @ Xuanwumen West Street, while 487 vehicles were captured at the intersection of Zaojunmiao Road @ Xueyuan South Road.

In this study, Adobe Premiere Pro software (Adobe Systems, San Jose, CA, USA) was also used to extract vehicles from the same video streams as those used by the VDCS and to verify the accuracy of the VDCS. The reason for using this software was because Adobe Premiere Pro is a professional video editing tool that provides better performance than manual counting [36]. Table 2 shows the comparison of the detection performance between the VDCS and Adobe Premiere Pro. The vehicle detection rate from the VDCS is 93.22% when compared with the “Ground-truth” case. “Ground-truth” means that vehicles are counted manually with Adobe Premiere Pro. “VDCS Detected” means that vehicles are counted by the VDCS. Additionally, the detection rates of speed and acceleration from the VDCS are 89.92% and 78.83%, respectively.

The dilemma zones for the intersection of Zaojunmiao Road and Xueyuan South Road are determined 40 m to 70 m away from the stop line, while the dilemma zones for the intersection of Naoshikou Street and Xuanwumen West Street are determined 50 m to 100 m away from the stop line. For the vehicles detected by the VDCS, 74% were observed with their drivers making “go” decisions, while 26% of their drivers opted to stop. This implies drivers’ misunderstanding of the “yellow signal” indication. Most drivers preferred to pass the intersection after the onset of the yellow signal, while they opted to stop only when faced with the high possibility of an RLR violation. Therefore, driver education would help reduce RLR violations and signal-related crashes.

The explanatory variables used for the analysis of drivers’ stop/go decisions and RLR violations are summarized in Table 3. V-TYPE is vehicle type (passenger car = 0, large-size vehicle = 1). DISTANCE, SPEED, and ACCELERATION are variables used to characterize vehicles approaching the intersections. DISTANCE, or yellow-onset distance, represents the distance from the current position of a vehicle to the stop line at the onset of the yellow signal. SPEED records the approaching speed of a vehicle at the onset of the yellow signal. ACCELERATION is the acceleration of a vehicle during the yellow interval, which is measured two seconds after the onset of the yellow signal. It is noted that the mean value of DISTANCE for vehicles with “stop” decisions (39.367 m) is longer than that of the ”go” decisions (20.317 m). For the drivers who made “go” decisions, DISTANCE for RLR violations (46.615 m) is longer than that of the drivers passing the intersections safely (19.850 m). The mean value of SPEED for the “go” decisions (39.492 km/h) is higher than that of the “stop” decisions (32.699 km/h). Besides, the mean value of ACCELERATION for the “go” decisions (0.342 m/s^2^) is positive, which indicates that most vehicles whose drivers made “go” decisions would accelerate to pass through the intersection.

It is also noted that a short yellow-onset distance and high approaching speed would encourage drivers to make ”go” decisions, and the aggressive “go” decisions with a relatively long yellow-onset distance and high acceleration would result in RLR violations.

## 4. Results and Discussion

A sequential logit model was developed to analyze the relationship between stop/go decisions and RLR violations. The results of the model are shown in Table 3. The Wald Chi-square statistic was applied to test variables’ significance in the sequential logit model. The goodness of fit was assessed by the Akaike Information Criterion (AIC) statistic, the Schwarz Criterion (SC) statistic, and the −2log-likelihood statistic. The following section presents the results of the model.

### 4.1. Model 1—The Stop/Go Decision Model

A driver’s correct stop/go decision at the onset of the yellow interval is essential for his or her vehicle to pass through an intersection safely. Inappropriate stops may result in rear-end crashes, while reckless “go” decisions may result in RLR violations and right-angle crashes. The data collected in the field surveys indicate that 341 drivers made “stop” decisions and 745 drivers made “go” decisions during the yellow interval.

As shown in Table 4, vehicle type, yellow onset distance and approaching speed are explanatory variables whose values met the 0.05 significant levels for entry into the logit model. The negative coefficient associated with the V-TYPE (OR = 0.519) implies that drivers in large-sized vehicles are 49.1% less likely to make “go” decisions than drivers in passenger cars during yellow intervals.

The results also indicate that the drivers with a relatively long yellow-onset distance can stop comfortably before the stop line. The odds ratio of the yellow-onset distance is 0.870, which means that if the vehicle’s distance to the intersection at the onset of the yellow signal increases by one unit (1 m), the probability of drivers making “go” decisions might decrease by 13%.

The positive coefficients of approaching speed indicate that drivers with high approaching speed are more likely to make “go” decisions. The odds ratio of the approaching speed is 1.144, which means that if the approaching speed increases by one unit (1 km/h), drivers would be 14.5% more likely to go through the intersection directly, while holding all other factors constant.

### 4.2. Model 2—The RLR Model

Drivers with go decisions may result in RLR violations. Among 745 drivers making “go” decisions, 13 RLR violations were observed.

As shown in Table 3, yellow-onset distance and acceleration are the explanatory variables in this model. The vehicle’s distance to the stop line at the onset of the yellow signal has a positive effect on RLR violations for the drivers with “go” decisions. If the yellow onset distance increases by one unit (1 m), the probability of drivers with RLR violations will increase by 9.6%. This result indicates that drivers with a long yellow-onset distance making “go” decisions are more likely to commit RLR violations. The positive coefficient of the acceleration suggests that the “go” drivers with higher acceleration are more likely to run red lights. The odds ratio of acceleration is 2.123, which reveals that if the acceleration increases by one unit (1 m/s^2^), drivers might be 2.123 times more likely to run red lights.

Table 2 illustrates the statistical survey of the drivers with “go” decisions. In the case of RLR violations, the mean value of the acceleration and yellow-onset distance is 0.998 m/s^2^ and 46.615 m, respectively. In the case of the vehicles crossing the intersection safely, the mean value of the acceleration is 0.323 m/s^2^, and the yellow-onset distance is 19.850 m, respectively. Aggressive drivers passing through the intersection with a long yellow onset distance and high acceleration during the yellow interval are more likely to run red lights.

### 4.3. Prediction Performance

The jackknife method was used to predict the classification of drivers’ behaviors through an iterative process. A subset of the extracted vehicles (or the extracted drivers) from the recorded video streams was created to calibrate the model, while the remainder of the extracted drivers was used in this study to train the model and predict drivers’ behaviors. This classification process was repeated until all the drivers had been classified.

The prediction performance of Models 1 and 2 is shown in Table 5. When the cutoff value selected in the classification process was set to 0.3, the correct, sensitivity and specificity values of Model 1 are 83.2%, 96.2%, and 65.3%, respectively, while the correct, sensitivity and specificity values of Model 2 are 96.1%, 98.8%, and 80.2%, respectively. The “correct” term shows the accuracy of the prediction. The higher the “correct” percentage, the higher the accuracy of the model for prediction. The “sensitivity” term presents the percentage of positives that are correctly identified or the true positive rate. The “specificity” term indicates the percentage of negatives that are correctly identified or the true negative rate.

**Figure 7:**
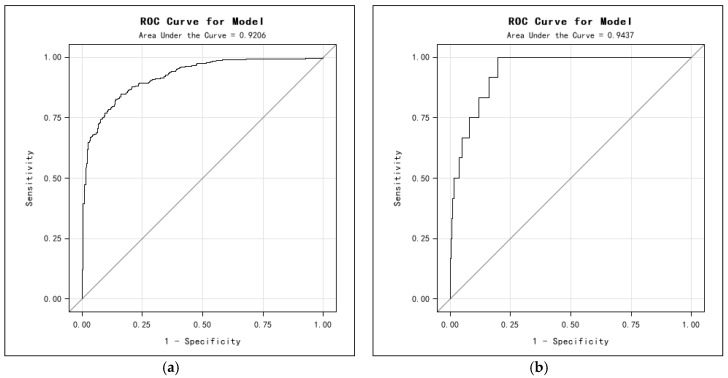
Receiver Operating Characteristic (ROC) curves. (**a**) Model 1; and (**b**) Model 2.

The predictive ability of a logit model is often evaluated in terms of its Receiver Operating Characteristic (ROC). In this study, a series of plotted pairs (1-specificity, sensitivity) was generated for Models 1 and 2 from different cutoff values from 0 to 1, as shown in Figure 7. The predictive accuracy for Models 1 and 2 measured by the area under the curve (AUC) is 0.921 and 0.944, respectively. The AUC results imply that the sequential logit model demonstrates good predictive performance for the prediction of stop/go decisions and RLR violations.

## 5. Conclusions

The use of the Vehicle Data Collection System (VDCS) for obtaining driver behaviors from recorded video streams and the application of a sequential logit model for analyzing and predicting driver behavior at signalized intersection are described in this paper.

A novel conceptual framework is proposed to study driver behavior related to the yellow interval. This new framework improves previous work [34,35,36,37,38], which used logit models to analyze drivers’ likelihood of passing intersections and running red lights separately. The sequential logit model used in this study offers a simple but effective way to model the relationship between drivers’ stop/go decisions and RLR violations. The model first depicts how explanatory variables affect stop/go decisions and then analyzes RLR violations caused by the “go” decisions.

The results of the model indicate that the yellow onset distance is an important predictor for both stop/go decisions and RLR violations. Drivers who travel a long distance to the stop line are more likely to make “stop” decisions when they approach intersections at the onset of the yellow signal. If those drivers opt to pass the intersections, the possibility of RLR violations is increased along with the travel distance to the stop line. This observation implies that aggressive drivers with “go” decisions may result in RLR violations. The possibility of RLR violations can be reduced by prolonging the time of the yellow interval; therefore, a stochastic yellow interval duration associated with intersection characteristics (e.g., roadway grade and speed limit) is recommended. With the development of sensor and communication technologies, dynamic dilemma zone protection systems can be applied to the target vehicles in the dilemma zone. One of the solutions is to extend the green signal to protect the target vehicles avoiding RLR violations. The other solution is to extend all-red signals at the intersections for the target vehicles, which can ensure the target vehicles pass through the intersections safely, while their RLR violations will be recorded. In addition, acceleration is also an important factor for RLR violations. Drivers who accelerate to cross the intersections are more likely to run red lights.

In summary, this study provides an effective framework to explore the relationship between drivers’ stop/go decisions and RLR violations. The findings of this study help traffic engineers understand the causes of RLR violations and aid in the development of appropriate countermeasures (such as driver assistance systems assisting drivers in making correct stop/go decisions) to avoid RLR violations.

## Figures and Tables

**Table 1:** Summary of intersection characteristics.

Surveyed Intersections	Through Lanes	Cycle Length (s)	Traffic Volume (vph)	Observed Samples
Naoshikou Street @ Xuanwumen West Street	3	190	1500	560
Zaojunmiao Road @ Xueyuan South Road	3	175	1200	526

**Table 2:** The comparison of detection performance.

Surveyed Intersections	Ground-Truth (Vehicles)	VDCS Detected (Vehicles)	Detection Rate (%)
Naoshikou Street @ Xuanwumen West Street	632	599	94.78
Zaojunmiao Road @ Xueyuan South Road	533	487	91.37
Total	1165	1086	93.22

**Table 3:** Vehicle information collected from the field surveys.

Variable	Description	Behavior	Mean	Standard Deviation	Minimum	Maximum
DISTANCE (m)	Vehicle Yellow-Onset Distance	Stop	39.367	13.364	3.000	80.000
		Go	20.317	14.751	0.000	82.000
		RLR	46.615	18.369	17.000	82.000
		Go Safely	19.850	14.262	0.000	70.000
SPEED (km/h)	Vehicle Approaching Speed at the Onset of Yellow Signal	Stop	32.699	11.056	11.560	67.500
		Go	39.492	11.757	12.000	72.000
		RLR	38.750	14.744	13.846	70.000
		Go Safely	39.505	11.710	12.000	72.000
ACCELERATION (m/s^2^)	Acceleration during the Yellow Interval	Stop	−2.363	1.059	−4.910	2.652
		Go	0.342	1.395	−2.688	3.571
		RLR	0.998	1.404	−2.688	3.571
		Go Safely	0.323	0.926	−0.490	2.500
V-TYPE (Vehicle Type)	Passenger Car = 1, Larger-Size Vehicle = 0					
Number of Stop Decisions: 341						
Number of Go Decisions: 745						
Number of RLR Violations: 13						
Number of Run Safely with Go Decisions: 732						

RLR = red light running.

**Table 4:** Estimation results for sequential logit model.

Stage	Variable	Coefficient	Standard Error	Wald Chi-Square	Odds Ratio (OR)	*p* Value
Model 1	V-TYPE	−0.655	0.189	12.017	0.519	0.001
	DISTANCE	−0.139	0.010	214.256	0.870	<0.0001
	SPEED	0.134	0.011	137.865	1.144	<0.0001
Model 2	DISTANCE	0.091	0.021	18.170	1.096	<0.0001
	ACCELERATION	0.753	0.192	15.260	2.123	<0.0001
-	Model 1		Model 2		-	-
-	Intercept Only	Intercept & Covariates	Intercept Only	Intercept & Covariates	-	-
AIC	1353.551	741.689	133.031	84.209	-	-
SC	1358.541	766.641	137.645	98.049	-	-
−2log-likelihood	1351.551	731.689	131.031	78.209	-	-

AIC = Akaike Information Criterion; SC = Schwarz Criterion.

**Table 5:** Model Prediction performance. AUC = area under the curve.

Models	Correct (%)	Sensitivity (%)	Specificity (%)	AUC
Model 1	83.2	96.2	65.3	0.921
Model 2	96.1	98.8	80.2	0.944
